# Central adjudication of serious adverse events did not affect trial’s safety results: Data from the Efficacy of Nitric Oxide in Stroke (ENOS) trial

**DOI:** 10.1371/journal.pone.0208142

**Published:** 2018-11-26

**Authors:** Peter J. Godolphin, Alan A. Montgomery, Lisa J. Woodhouse, Daniel Bereczki, Eivind Berge, Rónán Collins, Exuperio Díez-Tejedor, John Gommans, Kennedy R. Lees, Serefnur Ozturk, Stephen Phillips, Stuart Pocock, Kameshwar Prasad, Szabolcs Szatmari, Yongjun Wang, Philip M. Bath, Nikola Sprigg, on behalf of the ENOS Investigators

**Affiliations:** 1 Nottingham Clinical Trials Unit, University of Nottingham, Nottingham, United Kingdom; 2 Stroke Trials Unit, Division of Clinical Neuroscience, University of Nottingham, Nottingham, United Kingdom; 3 Department of Neurology, Semmelweis University, Budapest, Hungary; 4 Department of Internal Medicine, Oslo University Hospital, Oslo, Norway; 5 Tallaght Hospital, Dublin, Ireland; 6 Department of Neurology, La Paz University Hospital–Autonoma University of Madrid, Madrid, Spain; 7 Hawke’s Bay Hospital, Hastings, New Zealand; 8 University of Glasgow, Glasgow, United Kingdom; 9 Selcuk University Faculty of Medicine, Konya, Turkey; 10 Division of Neurology, Department of Medicine, Dalhousie University, Halifax, Canada; 11 London School of Hygiene and Tropical Medicine, London, United Kingdom; 12 All India Institute of Medical Sciences, New Delhi, India; 13 University of Medicine and Pharmacy, Targu Mures, Romania; 14 Tian Tian Hospital, Beijing, China; University Medical Center Gottingen, GERMANY

## Abstract

**Background and purpose:**

Central adjudication of serious adverse events (SAEs) can be undertaken in clinical trials, especially for open-label studies where outcome assessment may be at risk of bias. This study explored the effect of central adjudication of SAEs on the safety results of the Efficacy of Nitric Oxide in Stroke (ENOS) Trial.

**Methods:**

ENOS assigned patients with acute stroke at random to receive either transdermal glyceryl trinitrate (GTN) or no GTN and to Stop or Continue previous antihypertensive treatment. SAEs were reported by local investigators who were not blinded to treatment allocation. Central adjudicators, blinded to treatment allocation, reviewed the investigators reports and used evidence available to confirm or re-categorise the classification of event, likely causality, diagnosis and expectedness of event.

**Results:**

Of 4011 patients enrolled in ENOS, 1473 SAEs were reported by local investigators; this was reduced to 1444 after the review by adjudicators, with 29 re-classified as not an SAE. There was fair agreement between investigators and adjudicators regarding likely causality, with 808 agreements and 644 disagreements (56% crude agreement, weighted kappa, κ = 0.31). Agreement increased upon dichotomisation of the causality categories, with 1432 agreements and 20 disagreements (99% crude agreement, kappa = 0.54). Repeating the main trial safety analysis with investigator reported events showed that adjudication had no effect on the main trial safety conclusions.

**Conclusions:**

In a large trial, with many SAEs reported, central adjudication of these events did not affect trial conclusions. This suggests that adjudication of SAEs in a clinical trial where the intervention already has a well-established safety profile may not be necessary. Potential efficiency savings (financial, logistical) can be made through not adjudicating SAEs.

## Introduction

In clinical trials, the side-effects or hazards of an intervention must be measured alongside the potential benefits. Therefore, it is important that clinical trials collect high-quality data on the safety of an intervention as well as its potential efficacy. To ensure that data collected is reliable and robust, many clinical trials use a central adjudication process. This involves one or more experts who re-evaluate trial data, blinded to treatment assignment, to assess events reported by the local site assessors and decide whether or not they agree, or to categorise them in a specific way. Central adjudication is thought to improve the classification of events, therefore reducing noise and helping to improve accuracy and precision of effect estimates[1, 2]. For open-label studies, adjudication is also thought to help protect against detection bias, as adjudicators are blinded to treatment allocation (and patient characteristics) whenever possible[3]. It has been shown that unblinded local assessors often exaggerate effect estimates[4, 5]. FDA and EMA guidelines suggest that adjudication is particularly valuable ‘when the intervention is not delivered in a blinded fashion’ [6] or when `endpoints are complex to assess and/or include subjective components’[7].

The use of central adjudication in clinical trials has become commonplace in many clinical areas[8]. For example, over 80% of trials in cardiovascular disease are reported to include adjudication[9]. Both the FDA and EMA have published guidelines which advocate the need for an adjudication committee to assess cardiovascular outcomes in diabetes phase 2 and 3 trials[10, 11]. Cardiovascular safety outcomes are regularly adjudicated, with central adjudication often used to determine cause of death[12–14] and adverse events such as bleeding[15]. In an orthopaedic trial, self-reported cardiovascular serious adverse events such as stroke and myocardial infarction were adjudicated[16]. The study found that adjudication was vital to the trial results and interpretation, with only 58% of self-reported strokes verified by adjudicators. However, in a large stroke trial where adjudicators assessed cause of death, there was excellent agreement between adjudicators and local site investigators, with agreement being 94% and 93% for cardiovascular and cancer death respectively[14].

There is a current drive to reduce research waste in clinical trials, particularly through the REWARD campaign, which lists regulation and management[17] and efficient design, conduct and analysis[18] as areas to be addressed, as well as specifically targeting stroke research as an area to reduce waste through the European Stroke Organisation Trials Network Committee[19]. The campaign recommends that we ‘streamline and harmonise the laws, regulations, guidelines … and ensure that they are proportionate to the plausible risks associated with the research’ and calls for ‘additional research to learn how efficiency can be increased’[17]. Furthermore the campaign aims to “identify sources of waste in the way stroke research is chosen, designed, conducted, analysed, regulated, managed, disseminated, and reported”[19]. Given that adjudication of cardiovascular safety outcomes can be a burden to both the trial and site teams, taking considerable time and valuable resources to implement, it is important to assess the usefulness of this process.

The aim of this secondary analysis was to investigate the effect of central adjudication of serious adverse events (SAEs) on a large international stroke trial. The two objectives were to: (1) describe the level of agreement between central adjudicators and local investigators; (2) assess the effect of central adjudication of SAEs on the primary safety analysis of the trial.

## Methods

### Efficacy of Nitric Oxide in Stroke (ENOS) trial

The Efficacy of Nitric Oxide in Stroke (ENOS) trial was an international prospective randomised single-blind masked-endpoint partial-factorial trial that compared transdermal glyceryl trinitrate (GTN) with no GTN in patients with acute stroke and raised blood pressure[20]. A subset of patients who were already taking anti-hypertensive medication before entry into the trial were further assigned at random to continue or stop their medication. The trial recruited 4011 patients from 173 sites, across 23 countries on five continents between 2001 and 2013. The primary safety analysis found that there was no evidence to suggest the proportion of patients with SAEs differed between treatment groups. The protocol, statistical analysis plan, and main results for ENOS are described in detail elsewhere[20–22]. The clinical trial registration for ENOS is given on http://www.clinicaltrials.gov (Unique identifier: NCT00989716).

### Adjudication of investigator reported serious adverse events

Serious adverse events were defined in the protocol as adverse events that: were fatal, caused disability, were life threatening, led to hospital admission or prolonged discharge in a hospitalised patient, and/or resulted in birth defects in children born to trial participants. Excepting a small number of selected adverse events (such as headache), which were recorded but not adjudicated, non-serious adverse events were not recorded due to the established nature of the trial interventions. SAEs were recorded by local investigators using a web-based SAE form within 24 hours of the investigator being aware of the event. Local investigators were trained in SAE reporting before site initiation. Once five participants had been recruited by any participating trial site, there was a further site visit and ongoing data monitoring. Additional training was provided in yearly investigator meetings and regular trial newsletters also included hints and tips as well as frequently answered questions for SAE reporting.

It was not possible to blind local investigators to treatment allocation as they knew whether patients had received the GTN patch or not, and if patients were randomised to continue or stop their anti-hypertensive medication. The local investigator report included event classification, event diagnosis and evidence used to determine diagnosis, expectedness of event, and likely causality. Likely causality was reported in five categories: *Definitely not*, *Unlikely* (to be), *Possibly*, *Probably* and *Definitely* related to treatment.

Two central independent expert assessors, referred to throughout this article as adjudicators, who were blinded to treatment allocation, evaluated the investigator-reported SAEs. Each event was reviewed by a single adjudicator. The independent adjudicators had access to the web-based SAE form provided by the local investigator, a list of known adverse reactions and evidence provided by investigators including clinical notes, cranial scans, biochemistry and bacteriology data. Adjudicators were able to request further information from the specific trial sites if this was required to carry out the assessment of the SAE. Adjudicators confirmed or re-categorised investigators’ decisions on classification of event, event diagnosis, expectedness of event and likely causality. Any uncertainty was clarified by discussion with the chief investigator blinded to treatment allocation. The central adjudicator’s decision was treated as the gold standard for this study and was the decision used in all ENOS trial analyses.

### Statistical methods

Continuous variables were summarised using mean and standard deviation, or median and interquartile range. Categorical variables were described using number (%). Mann-Whitney u tests and Chi-square tests were used to determine imbalance between treatment groups for investigator reported SAEs. We used unweighted kappa to estimate agreement between local investigators and central adjudicators on event classification and diagnosis. Agreement between local investigators and central adjudicators on likely causality of SAEs was calculated using weighted kappa with symmetric weights. Weights were assigned as 1 if adjudicators and investigators agreed, as 0.75 if adjudicators and investigators causalities differed by one, as 0.5 if adjudicators and investigators causalities differed by two and 0 if adjudicators and investigators causalities differed by more than two. The likely causality categories were dichotomised into *Definitely not*, *Unlikely* and *Possibly* versus *Probably* and *Definitely*, with kappa used to estimate agreement. The number of patients with any SAE and the number of patients for each SAE diagnosis were compared by treatment arm using chi-squared tests for both local-investigator- and central-adjudicator-reported events. The time taken to adjudicate SAEs was estimated as the difference between the time the adjudicator first assessed the event and the time the event was completed. All analyses were performed in Stata version 14.0 or later.

## Results

Of 4011 patients enrolled into ENOS, 1031 (26%) were recorded as having had at least one SAE by local investigators. In total, local investigators reported 1473 SAEs, and of these, 1444 (98%) were classified as an SAE after assessment by central adjudicators.

The number of SAEs were similar between the GTN versus No GTN arms. Events were more likely to be moderate or mild than severe if a patient had been randomised to receive the GTN patch (Table 1). Investigators used clinical diagnostic evidence for roughly three-quarters of the SAEs (74%), with radiological (47%), biochemical (30%), haematological (26%) and ECG (23%) diagnostic evidence also used regularly. Adjudicators were certain of SAE diagnosis for the majority of investigator-reported events (76%), but indicated that their diagnosis was only possibly correct for 68 (5%) of the 1473 investigator-reported SAEs.

**Table 1:** Characteristics of investigator reported serious adverse events and certainty of adjudicator’s diagnosis.

	All	GTN	No GTN	p
**Patients**	4011	2000	2011	
**With SAE**	1031	522	509	
**SAEs**	1473	781	692	
**SAEs per patient**				
Mean (SD)	1.5 (1.0)	1.6 (1.1)	1.4 (0.8)	0.063*
Median [IQR]	1 [1, 2]	1 [1, 2]	1 [1, 2]	
Min, Max	1, 9	1, 9	1, 6	
**Timing of event**				
Before treatment	14 (1%)	6 (1%)	8 (1%)	0.61^†^
During treatment	642 (44%)	335 (43%)	307 (44%)	
After treatment	817 (55%)	440 (56%)	377 (54%)	
**Time from randomisation to event (days)**				
Mean (SD)	22.5 (27.1)	22.7 (28.6)	22.1 (25.5)	0.97*
Median [IQR]	11 [3, 34]	12 [3, 33]	10 [4, 36]	
Min, Max	0, 383	0, 383	0, 148	
**Diagnostic evidence used by investigators**^‡^				
Pathological	94 (6%)	45 (6%)	49 (7%)	0.74^†^
Radiological	685 (47%)	376 (48%)	309 (45%)	
ECG	336 (23%)	178 (23%)	158 (23%)	
Bacteriology	168 (11%)	93 (12%)	75 (11%)	
Biochemistry	443 (30%)	232 (30%)	211 (30%)	
Haematology	383 (26%)	201 (26%)	182 (26%)	
Clinical	1095 (74%)	593 (76%)	502 (73%)	
Other	234 (16%)	115 (15%)	119 (17%)	
**Intensity of event**				
Mild	214 (15%)	133 (17%)	81 (12%)	0.003^†^
Moderate	448 (30%)	246 (31%)	202 (29%)	
Severe	810 (55%)	402 (51%)	408 (69%)	
**Clinical outcome**				
Recovered	479 (33%)	268 (34%)	211 (30%)	0.006^†^
Not yet recovered	542 (37%)	302 (39%)	240 (35%)	
Died	451 (31%)	211 (27%)	240 (35%)	
**Certainty of adjudicators event diagnosis**				
Definite	1126 (76%)	593 (76%)	533 (77%)	0.062^†^
Probable	276 (19%)	153 (20%)	123 (18%)	
Possible	68 (5%)	34 (4%)	34 (5%)	

All data is N(%) unless otherwise indicated

GTN = Glyceryl trinitrate

*p-value from Mann-Whitney u test

^†^p-value from chi-square test

^‡^Categories not mutually exclusive

**Fig 1:**
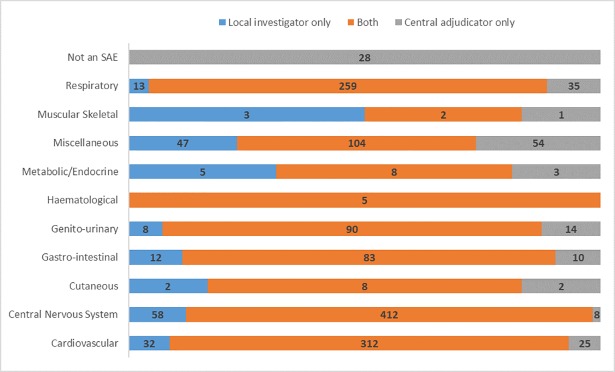
Agreement on serious adverse event diagnosis between central adjudicators and local investigators.

Agreement was excellent between central adjudicators and local investigators for the diagnosis of SAEs (Fig 1, *see Supporting Information*, S1 Table, crude agreement = 88%, unweighted kappa κ = 0.85). The highest number of disagreements, ignoring the miscellaneous category, was seen between cardiovascular events (25/337, 7%) and respiratory events (35/294, 12%). Genito-urinary SAEs had one of the largest percent disagreements for any category with over 20 events, with adjudicators disagreeing with investigators on 14/104 occasions (13%).

Within the cardiovascular category, there was excellent agreement (337 events, crude agreement = 84%, unweighted kappa, κ = 0.82). Agreement within the central nervous system was good (420 events, crude agreement = 76%, unweighted kappa, κ = 0.72). Within this category, investigators identified 57 (79%) of the 72 complication of initial stroke events, 75 (87%) of the 86 recurrent stroke events and 74 (73%) of the 102 extension of initial stroke events. A large number of the incorrectly identified events for these three sub-categories should have been classified in one of the other two sub-categories. For complication, recurrence and extension this crossover was 47%, 82% and 82% respectively. Agreement was only fair within the respiratory category (294 events, crude agreement = 73%, unweighted kappa, κ = 0.35), with only 192 of the 262 pneumonia events identified by investigators. 16 of these events were incorrectly classified as complication of initial stroke and 37 were classified by investigators as a chest infection.

There was fair agreement on likely causality of SAEs between central adjudicators and local investigators, with disagreement for 644/1452 (44%) events (Table 2, weighted kappa, κ = 0.31). When categories were dichotomised into *Definitely not*/*Unlikely/Possibly* versus *Probably*/*Definitely* there was near perfect agreement (Table 3, crude agreement = 99%, kappa κ = 0.54). If categories instead were dichotomised into *Definitely not*/*Unlikely* versus *Possibly*/ *Probably*/*Definitely*, then crude agreement remained high, but agreement over that expected by chance was reduced (crude agreement = 90%, kappa κ = 0.33).

**Table 2:** Agreement on likely causality of serious adverse events between central adjudicators and local investigators.

	Central Adjudicators					
Local Investigators	Definitely not	Unlikely	Possibly	Probably	Definitely	Total
Definitely not	558	154	28	0	0	740
Unlikely	325	223	47	1	0	596
Possibly	20	46	19	4	0	89
Probably	1	4	8	5	0	18
Definitely	0	0	2	4	3	9
**Total**	904	427	104	14	3	1452
**Disagreements (%)**	346 (38%)	204 (48%)	85 (82%)	9 (64%)	0 (0%)	644 (44%)

Crude agreement = 808/1452 = 56%

Weighted kappa = 0.31 (0.27, 0.35), Unweighted kappa = 0.20 (0.16, 0.24)

21 events did not have relatedness of treatment to SAE recorded by either adjudicators or investigators (Central Adjudicators = 19, Local Investigators = 2) and so were not included in this analysis

**Table 3:** Agreement on likely causality of SAEs between central adjudicators and local investigators–dichotomous analysis.

	Central Adjudicators		
Local Investigators	Definitely not, Unlikely or Possibly	Probably or Definitely	Total
Definitely not, Unlikely or Possibly	1420	5	1425
Probably or Definitely	15	12	27
**Total**	1435	17	1452
**Disagreements (%)**	15 (1%)	5 (29%)	20 (1%)

Crude agreement = 1432/1452 = 99%

Unweighted kappa = 0.54 (0.49, 0.24)

21 events did not have relatedness of treatment to SAE recorded by either adjudicators or investigators (Central Adjudicators = 19, Local Investigators = 2) and so were not included in this analysis

Central adjudication of SAEs had little impact compared with site investigators on the number of patients with any SAE reported for both the GTN vs No GTN and Continue vs Stop comparisons (Table 4). There were small differences between investigator- and adjudicator-reported SAEs for each SAE diagnosis, with evidence of a between-group effect differing for extension of initial stroke and recurrent stroke (both GTN vs No GTN comparison). Investigators poorly identified complication of initial stroke, with 57 events classified that were not confirmed by adjudicators (*see Supporting information*, S2 Table).

**Table 4:** Number of patients with serious adverse events during follow-up at day 90 classified by local investigators and central adjudicators: GTN vs no GTN and continue vs stop.

	GTN (n = 2000)	No GTN (n = 2011)	p-value*	Continue (n = 1053)	Stop (n = 1044)	p-value*
**Any SAE**						
Local Investigator	522	509	0.57	312	297	0.55
Central Adjudicator	520	502	0.45	310	294	0.52
**Complication of initial stroke**						
Local Investigator	67	53	0.18	26	36	0.19
Central Adjudicator	40	31	0.27	19	16	0.63
**Extension of initial stroke**						
Local Investigator	50	37	0.15	32	23	0.23
Central Adjudicator	59	37	0.021	33	23	0.19
**Symptomatic intracranial haemorrhage**						
Local Investigator	23	19	0.52	10	18	0.12
Central Adjudicator	27	17	0.13	11	16	0.32
**Recurrent stroke**						
Local Investigator	57	32	0.007	29	27	0.81
Central Adjudicator	47	35	0.17	25	27	0.80
**Myocardial infarction**						
Local Investigator	18	25	0.29	9	18	0.077
Central Adjudicator	19	22	0.65	11	16	0.32
**Sudden cardiac death**						
Local Investigator	3	5	0.48	1	3	0.31
Central Adjudicator	6	6	0.99	1	5	0.10
**Other cardiovascular event**						
Local Investigator	119	103	0.25	64	72	0.45
Central Adjudicator	109	97	0.37	60	66	0.55
**Pulmonary embolism**						
Local Investigator	26	17	0.16	10	11	0.81
Central Adjudicator	26	19	0.29	11	13	0.70
**Pneumonia**						
Local Investigator	89	100	0.44	74	50	0.030
Central Adjudicator	117	122	0.77	88	63	0.040
**Other event**						
Local Investigator	210	220	0.65	140	115	0.11
Central Adjudicator	212	212	0.95	134	119	0.35

*p-values are from a chi-squared test between treatment groups

GTN = Glyceryl trinitrate

Of the 1473 SAEs reported by local investigators, 15 had missing information for time of event completion. From the SAEs with complete data, 1297 (88%) were completed by adjudicators within 24 hours. 127 (9%) events took at least 90 days for the adjudication to be completed as they required further information that was not readily available.

## Discussion

Our study investigated the usefulness of central SAE adjudication in a large stroke trial with a single-blind design, hence local assessors were unbilnded to treatment allocation. We found that agreement between central adjudicators and local investigators was excellent for the diagnosis of events. However, there was worse agreement on likely causality of SAEs when assessed on a 5-point scale, although this improved substantially when categories were dichotomised. Overall, adjudication of SAEs had no impact on the primary safety results of ENOS, with a similar number of adjudicator-reported and investigator-reported SAEs reported.

Other studies have also reported that central adjudication has no effect on the primary safety results [15, 23]. However, one of these studies found that adjudication of specific SAE diagnoses (debilitating stroke, refractory angina, new heart failure) did alter the significance of these outcomes[23]. All of these diagnoses could be considered 'softer’ outcomes and rely on clinical factors and opinion rather than diagnostic tests and reports. These results are in agreement with our findings, which indicate that adjudication of SAEs has the potential to impact on the safety results of specific SAE diagnoses, in this case recurrent stroke and extension of initial stroke. However, caution should be taken with this result as in our study the number of events reported are low, the number of hypothesis tests conducted are high, and the actual number of events did not change dramatically in either case. In ENOS, adjudicators referred strictly to the protocol definitions for SAEs, whereas local investigators may not have followed these so rigorously, which could explain in part this finding. One potential approach for future studies could be to adjudicate a subset of events which are known to be difficult to diagnose, reducing the amount of adjudication needed whilst still ensuring the events with the highest chance of misclassification are adjudicated.

Furthermore, adjudication could be applied selectively, concentrating on major safety outcomes rather than all SAEs. Many trials, especially academically run, test treatments which already have a well-established safety profile, for which important safety issues can be predicted[24]. Therefore, relevant safety events can be listed at the trial design stage, and these can be chosen to be adjudicated, which would allow the number of events for adjudication to be greatly restricted and increase the chance that adjudication could be shown to impact meaningfully on the interpretation, since it limits the dilution of less relevant SAE ‘noise’.

We found reasonable agreement between adjudicators and investigators for likely causality of SAEs. Investigators were more likely to report that SAEs were related to treatment than central adjudicators. After dichotomisation, events could be categorised as serious adverse reactions (SARs) which would be SAEs rated as probably or definitely related to treatment. In this case, agreement increased to 99%, and there would have been few SAEs that would have been incorrectly categorised as serious adverse reactions (SARs) and vice versa. This implies that even though agreement on likely causality was not high, the disagreements were confined to the related/not related to treatment categories, and there was minimal crossover, suggesting adjudication of SAEs may not be needed to ensure SAEs and SARs are categorised properly. We found that the highest number of disagreements between adjudicators and investigators was for the *Possibly* category. Whilst assessing causality is subjective, the *Possibly* category is arguably the most subjective, and we propose that this category is currently unhelpful and not well understood. Furthermore, it could be argued that adjudicating causality is not relevant, given that safety reporting for serious adverse events in trials is required within a short pre-specified timeframe. Adjudication often takes place some weeks after the original event, and it is often the case that causality is not permitted to be downgraded, thus adjudication of causality has a limited impact in trials.

Whilst adjudication in trials may improve the accuracy of findings, the process comes at a considerable cost. We found that adjudicators required more information than that provided by investigators in 161 of the events they assessed, and this information took substantial time to obtain, in some cases with the adjudicators having to re-request data multiple times. Whilst only a portion of the time we estimated would have actually been used to adjudicate SAEs, this highlights the sizeable task of implementing adjudication in clinical trials. This burden is shared between central trial and site teams, and must be funded appropriately. In a thromboprophylaxis trial, adjudication was estimated to cost $75 per adjudicated event[25]. Therefore, using this as a rough guide due to similarities in adjudication methods, we could assume that adjudication of SAEs in ENOS may have cost in the region of $110,000. This amount is not insignificant, and, if adjudication of SAEs continues to be prescribed as best practice, or even a necessity for trials that investigate cardiovascular outcomes, then the cost of such a process needs to be properly evaluated against the benefits that it actually brings to a trial.

A strength of this study is that we had data on nearly 1500 SAEs from a large, well conducted randomised trial to use in our analysis. As well as this, the amount of missing data was low, with a high level of data completeness for both adjudicator- and investigator-assessed events. This study was designed and undertaken once the ENOS trial had finished, and so it was not possible that the presence of this study had any influence on investigators’ or adjudicators’ behaviour. One limitation of this study was that there was no quality control measure due to each SAE only being assessed by a single adjudicator. Whilst this meant the process used less resources, there was also a higher chance of random error[26], but level of agreement suggests this wasn’t an issue in ENOS. Additionally, in ENOS, only events reported by investigators were adjudicated. Thus, after adjudication, the total number of SAEs could either stay the same, or decrease. This approach could potentially miss SAEs that were not identified by investigators during the trial. In fact, as investigators were not blinded to treatment allocation, there was the potential for SAEs to be selectively reported. Granger et al.[1] suggests a potential alternative, where adjudicators also review “events that are ‘triggered’ by some feature that raises the suspicion of an event”. This method has been carried out previously, with computer algorithms used to identify events to adjudicate[8].

In summary, this study found that local site investigators could accurately determine SAE diagnosis in a large international trial. Adjudication of investigator-reported SAEs had no impact on the main trial safety conclusions. The purpose of SAE adjudication in a large trial, where the intervention already has a well-established safety profile, needs to be considered before implementing a potentially expensive and time-consuming process. Further research should provide an accurate evaluation of the cost and time involved in adjudication to better understand how useful the process is for clinical trials.

## Supporting information

S1 Table Agreement between local investigators and central adjudicators on the number of patients with serious adverse events during follow-up at day 90. (DOCX)

S2 Table Agreement between local investigators and central adjudicators on the number of patients with serious adverse events during follow-up at day 90. (DOCX)
