# Supplementary material for: Central adjudication of serious adverse events did not affect trial’s safety results: Data from the Efficacy of Nitric Oxide in Stroke (ENOS) trial
Source: PLoS One. 2018 Nov 26;13(11):e0208142. doi: 10.1371/journal.pone.0208142 (PMC6258247; doi:10.1371/journal.pone.0208142)
Supplement: S1 Table — (DOCX) [file pone.0208142.s001.docx]

**S1 Table:** Agreement on Diagnosis of serious adverse events between central adjudicators and local investigators

|  | **Central Adjudicators** | | | | | | | | | | | |
| --- | --- | --- | --- | --- | --- | --- | --- | --- | --- | --- | --- | --- |
| **Local Investigators** | Cardiovascular | Central Nervous System | Cutaneous | Gastro-intestinal | Genito-urinary | Haematological | Metabolic / Endocrine | Miscellaneous | Muscular Skeletal | Respiratory | Not an SAE | **Total** |
| Cardiovascular | 312 | 3 | 0 | 0 | 0 | 0 | 0 | 14 | 0 | 7 | 8 | 344 |
| Central Nervous System | 8 | 412 | 0 | 3 | 3 | 0 | 1 | 15 | 0 | 18 | 10 | 470 |
| Cutaneous | 0 | 0 | 8 | 0 | 0 | 0 | 0 | 2 | 0 | 0 | 0 | 10 |
| Gastro-intestinal | 0 | 0 | 0 | 83 | 0 | 0 | 0 | 7 | 0 | 1 | 4 | 95 |
| Genito-urinary | 0 | 0 | 1 | 0 | 90 | 0 | 0 | 5 | 0 | 1 | 1 | 98 |
| Haematological | 0 | 0 | 0 | 0 | 0 | 5 | 0 | 0 | 0 | 0 | 0 | 5 |
| Metabolic / Endocrine | 0 | 0 | 0 | 0 | 1 | 0 | 8 | 2 | 0 | 1 | 1 | 13 |
| Miscellaneous | 12 | 5 | 1 | 7 | 10 | 0 | 2 | 104 | 1 | 7 | 2 | 151 |
| Muscular Skeletal | 0 | 0 | 0 | 0 | 0 | 0 | 0 | 2 | 2 | 0 | 1 | 5 |
| Respiratory | 5 | 0 | 0 | 0 | 0 | 0 | 0 | 7 | 0 | 259 | 1 | 272 |
| Not an SAE | 0 | 0 | 0 | 0 | 0 | 0 | 0 | 0 | 0 | 0 | 0 | 0 |
| **Total** | 337 | 420 | 10 | 93 | 104 | 5 | 11 | 158 | 3 | 294 | 28 | 1463 |
| **Disagreements (%)** | 25 (7%) | 8 (2%) | 2 (20%) | 10 (11%) | 14 (13%) | 0 (0%) | 3 (27%) | 54 (34%) | 1 (33%) | 35 (12%) | 28 (100%) | 180 (12%) |

Crude agreement = 1283/1463 = 88%

Unweighted kappa = 0.85 (0.82, 0.87)

Local Investigators did not record diagnosis of SAE for 10 events and so could these were not included in this analysis
