# Supplementary material for: Central adjudication of serious adverse events did not affect trial’s safety results: Data from the Efficacy of Nitric Oxide in Stroke (ENOS) trial
Source: PLoS One. 2018 Nov 26;13(11):e0208142. doi: 10.1371/journal.pone.0208142 (PMC6258247; doi:10.1371/journal.pone.0208142)
Supplement: S2 Table — (DOCX) [file pone.0208142.s002.docx]

**S2 Table:** Agreement between local investigators and central adjudicators on the number of patients with serious adverse events during follow-up at day 90

|  | **All (n=4011)** | **GTN (n=2000)** | **No GTN (n=2011)** | **Continue (n=1053)** | **Stop (n=1044)** |
| --- | --- | --- | --- | --- | --- |
| **Any SAE** |  |  |  |  |  |
| Both classify event | 1022 | 520 | 502 | 310 | 294 |
| Local investigators classify event only | 9 | 2 | 7 | 2 | 3 |
| Central adjudicators classify event only | 0 | 0 | 0 | 0 | 0 |
| **Complication of initial stroke** |  |  |  |  |  |
| Both classify event | 57 | 33 | 24 | 13 | 13 |
| Local investigators classify event only | 57 | 31 | 26 | 12 | 20 |
| Central adjudicators classify event only | 14 | 7 | 7 | 6 | 3 |
| **Extension of initial stroke** |  |  |  |  |  |
| Both classify event | 68 | 43 | 25 | 25 | 17 |
| Local investigators classify event only | 16 | 5 | 11 | 5 | 5 |
| Central adjudicators classify event only | 27 | 15 | 12 | 8 | 5 |
| **Symptomatic intracranial haemorrhage** |  |  |  |  |  |
| Both classify event | 34 | 19 | 15 | 10 | 14 |
| Local investigators classify event only | 6 | 3 | 3 | 0 | 3 |
| Central adjudicator classify event only | 7 | 6 | 1 | 1 | 1 |
| **Recurrent stroke** |  |  |  |  |  |
| Both classify event | 70 | 41 | 29 | 21 | 23 |
| Local investigators classify event only | 14 | 11 | 3 | 7 | 3 |
| Central adjudicators classify event only | 10 | 4 | 6 | 4 | 3 |
| **Myocardial infarction** |  |  |  |  |  |
| Both classify event | 33 | 12 | 21 | 9 | 13 |
| Local investigators classify event only | 7 | 3 | 4 | 0 | 4 |
| Central adjudicators classify event only | 6 | 5 | 1 | 2 | 2 |
| **Sudden cardiac death** |  |  |  |  |  |
| Both classify event | 4 | 2 | 2 | 0 | 2 |
| Local investigators classify event only | 3 | 1 | 2 | 1 | 0 |
| Central adjudicators classify event only | 8 | 4 | 4 | 1 | 3 |
| **Other cardiovascular event** |  |  |  |  |  |
| Both classify event | 160 | 87 | 73 | 47 | 48 |
| Local investigators classify event only | 24 | 11 | 13 | 8 | 7 |
| Central adjudicators classify event only | 14 | 5 | 9 | 6 | 3 |
| **Pulmonary embolism** |  |  |  |  |  |
| Both classify event | 30 | 19 | 11 | 7 | 7 |
| Local investigators classify event only | 0 | 0 | 0 | 0 | 0 |
| Central adjudicators classify event only | 5 | 1 | 4 | 1 | 3 |
| **Pneumonia** |  |  |  |  |  |
| Both classify event | 139 | 61 | 78 | 52 | 37 |
| Local investigators classify event only | 5 | 1 | 4 | 4 | 1 |
| Central adjudicators classify event only | 42 | 24 | 18 | 10 | 12 |
| **Other event** |  |  |  |  |  |
| Both classify event | 246 | 112 | 134 | 74 | 67 |
| Local investigators classify event only | 50 | 26 | 24 | 14 | 11 |
| Central adjudicators classify event only | 48 | 20 | 28 | 13 | 18 |

GTN = Glyceryl trinitrate
